# Enhancing the Inherent Flame Retardancy of Polylactic Acid by Anchoring Phytic Acid-Lysine Using Epoxidized Tannic Acid

**DOI:** 10.1021/acsapm.5c03450

**Published:** 2025-12-02

**Authors:** Jazmine Aiya D. Marquez, Wan Zhang, Navaporn Suphavilai, Manish Shetty, Hae-Kwon Jeong, Qingsheng Wang

**Affiliations:** Artie McFerrin Department of Chemical Engineering, 14736 Texas A&M University, College Station, Texas 77843, United States

**Keywords:** polylactic acid, flame retardant, biopolymer, phytic acid, tannic acid

## Abstract

Inherent flammability is a major limitation of polylactic acid (PLA) in industrial applications. Several flame retardants (FRs) have been explored to address this, but many undergo complicated synthesis routes, often accompanied by toxic chemical use. Moreover, these FR systems negatively affect the biodegradability of PLA. In this study, epoxidized tannic acid (ETA) and a phytic acid-lysine (PALys) biobased FR were used. The former has two functions: an anchor and a carbon source, while the latter functions as a synergistic phosphorus–nitrogen (P–N) FR. The anchoring process of PALys to the PLA end groups using ETA was accomplished through melt blending. The heat and shear allow the epoxy groups in ETA to react to OH groups in PALys and PLA. The addition of 1 wt % ETA and 5 wt % PALys improved PLA thermal stability by reducing the weight loss rate from 40.69 to 2.99 wt %/min. Moreover, microscale combustion calorimeter (MCC), limiting oxygen index (LOI), UL-94, and cone calorimeter tests were performed to evaluate the efficacy of the FR system. PLA/1ETA/5PALys achieved a high LOI value of 34% vol, a UL-94 V-0 rating, and 50% reduction in the flame out time. Char formation was also observed during the combustion tests. Additionally, the FR mechanism was evaluated using scanning electron microscopy–energy-dispersive spectroscopy and thermogravimetry–mass spectrometry. The latter established the gas-phase action by the emission of noncombustible gases, such as ammonia, CO_2_, and H_2_O, from the decomposition of lysine and ETA, which reduces the combustible gas concentration. Furthermore, phosphorus from PALys was able to dehydrate ETA and PLA to catalyze the formation of stable aromatic and phosphorus-containing char.

## Graphical abstract

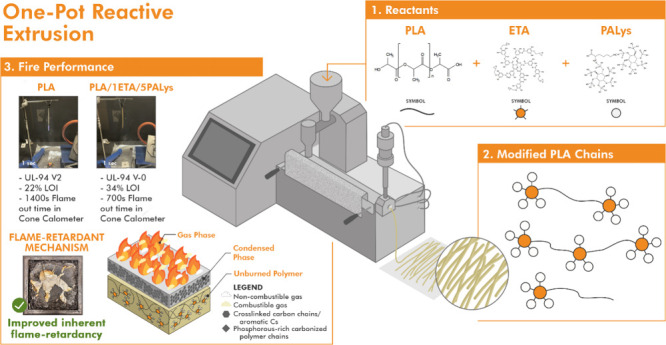

## Introduction

Polylactic acid (PLA) is a biomass-sourced, biodegradable, and biocompatible aliphatic semicrystalline polymer that has the potential to replace petroleum-based polyolefins because of its high modulus and strength, as well as its capability to be processed using different processing techniques.^1,2^ Moreover, the shift to sustainable polymers has driven the demand for PLA with a 1.5 billion USD market size in 2023 and a forecasted compound annual growth rate of 17.1% until 2028.^3^ However, it is not without challenges; one significant concern is its inherent flammability.^4^ PLA, in the presence of a flame, produces molten droplets that ignite, drip, and become a secondary source of ignition. Hence, it is imperative that the inherent flammability of PLA be addressed to broaden its potential uses.

FRs are used to improve PLA flammability and reduce its fire hazard potential. Phosphorus-containing FRs have been widely investigated because of their excellent compatibility with PLA.^5^ They can function as a “flame poison” by scavenging active hydrogen and hydroxyl radicals during pyrolysis and suffocate the flame to prevent further combustion.^6^ On the other hand, they can also dehydrate the PLA backbone and promote carbonization to form char.^7^ However, the aliphatic nature of the PLA backbone prevents it from forming a fully cross-linked structure during combustion. As a result, the char formed with the help of phosphorus-containing FRs is also susceptible to char oxidation that eventually volatilizes when continuously exposed to high temperatures. With this, phosphorus-containing FRs are used in conjunction with a carbon source to enhance their char forming capabilities^8^ and a nitrogen-containing compound to assist in diluting the gas phase of combustible compounds.^9^ Although there is an abundance of possible P–N–C FR combinations that have been investigated, the synthesis is relatively complex. The new generation of PLA FR should be simple in synthesis, as well as nontoxic and hazardous waste-free.^10^ Moreover, these FRs should have good compatibility with PLA to preserve its biodegradable nature.^11^

Phytic acid (PA) is a phosphorus storage molecule found in plants that are known to promote mineral deficiencies in humans. It is a 6-fold dihydrogen phosphate ester of inositol that has been proven to function as a condensed-phase flame retardant for PLA.^12–14^ To improve its FR efficacy, it is usually modified with a nitrogen-containing compound that functions as a diluting agent. Amino acids are nitrogen-containing compounds that are great diluting agents during combustion because they can release nonflammable gas. They are also biobased, which are naturally occurring in the environment and nutrient-rich, preserving the biodegradability of PLA. Moreover, the process of binding the amino acid onto PA is simple and nontoxic, which makes it an attractive alternative to conventional P–N FRs.^15^ Several studies have also proven its effectiveness in various polymeric systems. Xu et al.^16^ have achieved a UL-94 V-0 rating and an LOI of 26.8% vol with the addition of a 1 wt % PA-arginine FR in PLA, as well as improved crystallization. Gao et al.^12^ proved that 18 wt % addition of a PA-piperazine FR enhances the LOI of polypropylene by 38.9% with a V-0 UL-94 rating and reduced its smoke production. The investigation of Kong et al.^17^ showed that the combination of PA-arginine and hydroxylated montmorillonite has a synergistic effect on the flame retardancy of polybutylene succinate. Leng et al.^18^ also proved that the PA-tyramine FR works as an FR for wood-plastic composites, which shows the versatility of the FR. In this study, lysine was used as the N-containing FR to work synergistically with PA. It is a diamino acid that is usually found in beans and plants that has been proven to function well as a gas-phase FR.^19^ Cheng et al.^20^ used lysine together with ammonium polyphosphate in an epoxy resin where the former improved the intumescence of epoxy, leading to a better FR performance. The PA and lysine combination has shown synergistic effects on improving the FR properties of cellulose-based fabrics.^21^ Moreover, this P–N combination can produce high-strength char and an excellent dehydrating process in a polypropylene system compared to arginine and histidine.^22^ Hence, this study will investigate the effectiveness of the PALys FR in a PLA system.

Tannic acid (TA) is an abundant biobased polyphenol that is extracted from tannins.^23^ It is made up of two layers of gallic acid units with a glucose ring core that has high functionality.^24^ Upon combustion, TA can function in both the gas and condensed phase. For the former, it releases radical scavengers in the form of 1,2,3-benzene triol^25^ that can provide protons to free radicals and create a quenching effect.^26,27^ On the other hand, the key condensed-phase action to function as a carbon source improves the polymer’s charring ability. To address the lack of cross-linked or aromatic structures in PLA after burning, TA was used as a carbon source. In this study, TA was epoxidized to function not only as a carbon source but also as an anchor for PALys and PLA. PALys is considered a small-molecule FR, which can eventually leach out of the PLA matrix. On the other hand, ETA has been widely explored as an FR for thermosetting polymers.^28,29^ By using ETA as an anchor to the PLA end groups, we could preserve the effectiveness of the FR. Moreover, the anchoring process can be achieved using melt blending because the epoxy groups of TA and the OH groups of PLA and PALys can readily react in the presence of heat and shear.^30^ Thus, the objectives of this study are to synthesize the biobased additives PALys and ETA, perform melt blending using a twin-screw extruder to anchor ETA and PALys to the PLA end groups, assess the flame-retardant capability of the PLA/ETA/PAlys system, and propose an FR mechanism that best describes the FR system.

## Methodologies

### Materials

PLA (4032D) was obtained from NatureWorks Corp. (USA) in a pellet form. The weight-average molecular weight (*M*_w_) is 180 kDa, and the *M*_w_/*M*_n_ ratio is 1.5. Aqueous PA solution (50% w/w) was procured from Sigma-Aldrich (St. Louis, MO, USA). l-Lysine (98%), epichlorohydrin (ECH), and TA were purchased from Thermo Scientific (Waltham, MA, USA). Tetrahydrofuran (THF) and 5 and 0.5 equiv/L sodium hydroxide (NaOH) were supplied by Fisher Chemical (Pittsburgh, PA, USA). Ethanol was acquired from Reagents (Belmont, NC, USA).

### PALys Synthesis

PALys was synthesized using a 1:3 molar ratio of 50 wt % PA (3.15 g) and lysine (1.05 g).^22^ The former was added to 50 mL of deionized water and sonicated for 10 min to promote the molecular movement of PA. Afterward, lysine was mixed into the solution using a magnetic stirrer. It was stirred for 30 min, allowing the reaction to take place. Following this step, the solution was slowly poured into 250 mL of ethanol with stirring to precipitate the PALys salt. The solid was then allowed to decant for 1 day before collecting it. Subsequently, it was dried in a vacuum oven at 60 °C for 18 h and was crushed and grinded before it was stored for further use.

### ETA Synthesis

The ETA synthesis was based on the study of Borah and Karak.^31^ In a double-neck round-bottom flask, 10 g of tannic acid was combined with 15 mL of 0.5 equiv/L NaOH using a magnetic stirrer set to 200 rpm. The temperature was then set to 80 °C. When the temperature reached 40 °C, 23 mL of ECH was added dropwise. Similarly, 50 mL of 5 equiv/L NaOH was slowly added (dropwise) into the solution starting at 65 °C, to control the pH. The solution was continuously stirred for 2–3 h at 80 °C, allowing the solution to react. Afterward, NaOH addition was ceased, and the solution was allowed to stir for another 1 h at 80 °C. It was then cooled to room temperature. The liquid portion was removed, and the solid was washed with deionized water 3 times, followed by washing using NaCl solution 3 times. The solid was then dissolved in THF. A rotary evaporator was used to remove most of the solvent, followed by drying in a vacuum oven at 80 °C for 24 h. The dried solids were crushed and grinded, followed by storage for further use.

### PLA/ETA/PALys Extrusion

PLA pellets were dried in a vacuum oven at 80 °C for 12 h before melt blending. The extrusion of PLA and its composites was performed by using a twin-screw extruder (TSE) (Process 11 Parallel TSE, Thermo Fisher Scientific).^32^ The screws had an L/D ratio of 40 and a diameter of 11 mm. PLA and the PLA dry blend—PLA mixed with FR additives through shaking—were flood fed at a rate of 5.8 g/min. The temperature profiles used for melt compounding were 160, 180, 185, 185, 190, 190, 190, and 190 °C with a motor rotation speed of 60 rpm. After extrusion, the polymer was cut into pellets and stored until testing. Table 1 lists the naming convention and polymer composition used in this study.

**Table 1:** Composition of PLA Composites

	wt %		
sample name	PLA	PALys	ETA
PLA	100	0	0
PLA/5PALys	95	5	0
PLA/1ETA	99	0	1
PLA/1ETA/1PALys	98	1	1
PLA/1ETA/5PALys	94	5	1
PLA/5ETA/5PALys	90	5	5

### Characterization

The functional groups of the modified biobased additives were identified using an attenuated total reflectance–Fourier transform infrared (ATR-FTIR) spectrometer Thermo Scientific Nicolet iS5 equipped with an iD7 ATR (Waltham, MA, USA). The instrument was set to a resolution of 4 with 50 scans. A TGA Q50 from TA Instruments (New Castle, DE, USA) was used to determine the thermal stability of PLA and its composites in air. The test used a heating rate of 10 °C/min, which increases from 30 to 700 °C. The exhaust gases from the TGA were connected using a capillary tube to the inlet of a Stanford Research Systems UGA200 Universal Gas Analyzer (UGA; Sunnyvale, CA, USA) equipped with a mass spectrometer. The UGA collects pressure versus time scans at a 2 s interval during the temperature-programmed oxidation phase of the TGA. The sample was scanned for CO_2_ (44 amu), H_2_O (18 amu), and ammonia (17 and 15 amu) gas release. Images of the char and its elemental composition after cone testing were taken by a JEOL JSM-7500F (Peabody, MA, USA), a field-emission scanning electron microscope (FE-SEM) equipped with energy-dispersive X-ray spectroscopy (EDS), with an acceleration voltage of 5 kV. The ^13^C NMR spectra were recorded on a 10 kHz Avance Neo 400 solid-state NMR (Bremen, Germany).

### Fire Performance Testing

The microscale combustion calorimeter (MCC) (Fire Testing Technology, UK) used for polymer screening is a type of pyrolysis-combustion flow calorimetry where the sample was initially decomposed under anaerobic conditions, mimicking realistic fire behavior in which the material undergoes pyrolysis to decompose the polymer. To simulate this environment, a nitrogen gas stream flowing at a rate of 80 cm^3^/min was allowed to enter the chamber containing a sample of ∼3 mg that was heated from 150 to 650 °C at a heating rate of 1 °C/s. Upon thermal decomposition, the pyrolysis products were mixed with 20 cm^3^/min oxygen before entering the combustor that was set at 900 °C, where they will completely oxidize. The results were processed using MCC curve fit 17 software (Fire Testing Technology, UK). The limiting oxygen index (LOI) was ascertained using an FTT oxygen index apparatus (Fire Testing Technology, UK) following ASTM D2863, using a specimen size of 125 mm × 6.5 mm × 3.2 mm (*L* × *W* × *H*). A horizontal/vertical flame chamber (Fire Testing Technology, UK) was utilized to determine the UL-94 vertical combustion rating based on ASTM D3801. The samples were molded to have a dimension of 130 mm × 13 mm × 3.2 mm. An iCone classic calorimeter (Fire Testing Technology, UK) was employed to determine the combustion performance of the samples based on ASTM E1354. This test exposes the surface of a sample, with dimensions of 100 mm × 100 mm × 3.2 mm, to an irradiance heat flux of 50 kW/m^2^, which exemplifies a severe fire exposure comparable to real fires, to determine the reaction-to-fire properties of PLA and its composites under well-ventilated and forced-combustion conditions. The samples were ignited using a spark igniter until sustained combustion was achieved.^33^

## Results and Discussion

### Chemical Structure Analysis of the Bioadditives

Figure 1 depicts the confirmation of ETA and PALys synthesis. The ATR-FTIR curve for TA in Figure 1a illustrates the presence of the characteristic peaks of TA at 1606, 1705, and 3020 cm^–1^ representing the C=C stretching, C=O stretching, and OH stretching, respectively. Upon epoxidation, the OH stretching peak sharpened and shifted to the left at 3380 cm^–1^, with the appearance of the C–H stretching peaks at 2939 and 2577 cm^–1^, as well as the oxirane ring peak at 905 cm^–1^. Moreover, the ^13^C NMR comparison between TA and ETA is shown in Figure 1b. It confirmed the presence of TA characteristic peaks at 167 (a), 145 (b), 139 (c), 120 (d), 112 (e), and 72 (f) ppm corresponding to carbonyl Cs of TA, benzene ring Cs attached to −OH groups, benzene ring Cs next to C–O, benzene ring Cs next to C=O, unsubstituted benzene ring Cs, and Cs in the central glucose ring, respectively. On the other hand, the ^13^C NMR curve for ETA presented new peaks at 52 and 46 (h and i) ppm that represent the Cs of the oxirane ring, as well as a peak at 145 (g) ppm representing the benzene ring Cs next to C–O that connects the epoxy group to the TA molecule. There is an increase in intensity at the 72 (f) ppm chemical shift from the overlap of the peak, which represents the Cs next to the oxirane and the Cs from the glucose core. Moreover, peaks for the benzene ring meta carbon next to the C–O bond shifted to 149 (c) and the Cs attached to the −OH groups shifted to 153 (b). These are consistent with the work of Boro and Karak^28^ and Borah and Karak,^31^ which confirms the formation of ETA.

**Figure 1:**
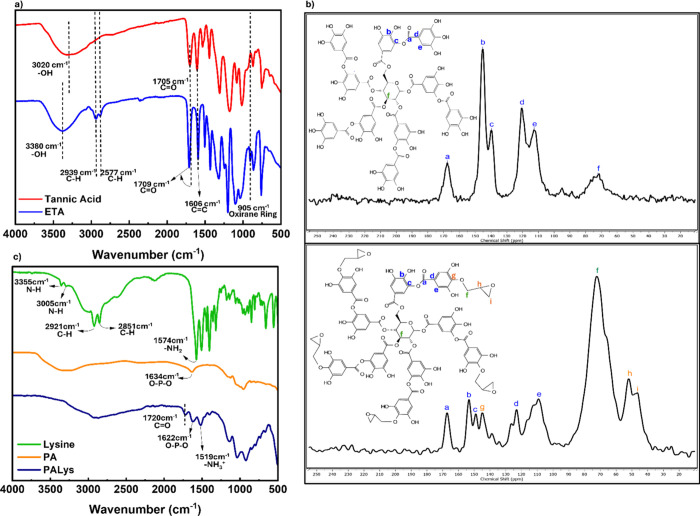
(a) TA and ETA FTIR curve, (b) ^13^C NMR comparison of TA and ETA, and (c) FTIR curves of lysine, PA, and PALys.

Figure 1c shows the ATR-FTIR characterization of PALys. The C–H stretching of lysine around 2851 and 2921 cm^–1^ overlapped with the broad peak between 2250 and 3500 cm^–1^ after the reaction with PA because of the increased presence of functional groups with hydrogen bonding, such as carboxylic acid from lysine plus the −OH groups from PA. A peak at 1720 cm^–1^ related to the C=O stretching also appeared.^34^ Moreover, the O–P–O stretching of PA at 1634 cm^–1^ and −NH_2_ bending of lysine at 1574 cm^–1^ shifted to the right at wavelengths of 1622 and 1519 cm^–1^, respectively. The shift of the O–P–O peak to 1622 cm^–1^ could be attributed to the low concentration of PA in PALys. On the other hand, the shift of the primary amine bending peak to 1519 cm^–1^ is indicative of weakened N–H bonds due to protonation of the primary amine, forming −NH_3_^+^. Furthermore, the ionic bond between −NH_3_^+^ of lysine and −O^–^ of PA resulted in the disappearance of amine stretching peaks at 3005 and 3355 cm^–1^ because of the strong electrostatic attraction that holds the two compounds together in a crystal lattice, preventing the protonated amine from stretching. This signifies the successful bonding of PA and lysine; the structure of PALys is shown in Figure S1.

### Anchoring ETA and PALys onto PLA

Figure 2 illustrates the FTIR curves to confirm the attachment of ETA and PALys to the PLA backbone. From Figure 2a, the characteristic peaks of PLA at 2993, 2964, and 2919 cm^–1^ represent the symmetric C–H stretching; 2850 cm^–1^ presents the asymmetric C–H stretching; 1750 cm^–1^ indicates the presence of C=O; the 1360–1452 cm^–1^ range is designated for the −CH­(CH_3_) bond; the range of 1050–1250 cm^–1^ signifies the presence of C–O from carboxyl groups and C–O–C vibration; 1077 and 1042 cm^–1^ may be attributed to the C–H vibrations in CH_3_; and 866 and 754 cm^–1^ represent the O–CH–CH_3_ and α-CH wagging present, respectively.^35–37^ Upon the addition of 1 wt % ETA (PLA/1ETA), the oxirane ring at 905 cm^–1^ disappeared, as illustrated at Figure 2b, which indicates that the epoxy groups of ETA had reacted with the OH end groups of PLA. The peak at 2919 cm^–1^ also became broader for PLA/1ETA that signifies the presence of C–H groups from ETA. For PLA/5PALys, a broad curve at 942 cm^–1^ was observed (refer to Figure 2b), which represents the P–O–C bond of PA. Moreover, the peak at 2919 cm^–1^ appears to have a similar intensity to the 2945 cm^–1^ peak, which could be attributed to the aliphatic chain of lysine in PALys. In compounding PLA/1ETA/5PALys, the oxirane ring at 905 cm^–1^ also disappears. This indicates its reaction with the OH groups of either PLA or PALys. A peak at 942 cm^–1^ was observed, confirming the presence of phosphorus groups from PALys. Furthermore, Figure 2c illustrates a closer look at the 1500–2000 cm^–1^ range. A shoulder peak for PLA/1ETA and PLA/1ETA/5PALys was noticed. It depicts the presence of C=O stretching from ETA. Figure 2d exhibits the peak in the baseline-corrected 2890–2980 cm^–1^ range. The 2919 cm^–1^ peak shifted by 3 cm^–1^ to the left (2922 cm^–1^) with an increase in broadness, which is an indication of structural modification of the oxirane C–H groups after the reaction with OH groups.^38,39^ Table S1 provides a quantitative comparison to confirm the peak broadening of PLA and the modified PLA samples. Contrary to PLA/1ETA where only peak broadening was observed, the peak broadening and shift for PLA/1ETA/5PALys signify that the structural rearrangement comes from the reaction with both PLA and PALys, leading to differences in conformational disorder between the PLA/ETA and the ETA/PALys bonds.^40^ Further confirmation of the successful anchoring of PALys through ETA using ^1^H NMR can be found in Figures S3 and S4. Hence, the attachment of ETA and PALys to the ends of the PLA chain was confirmed.

**Figure 2:**
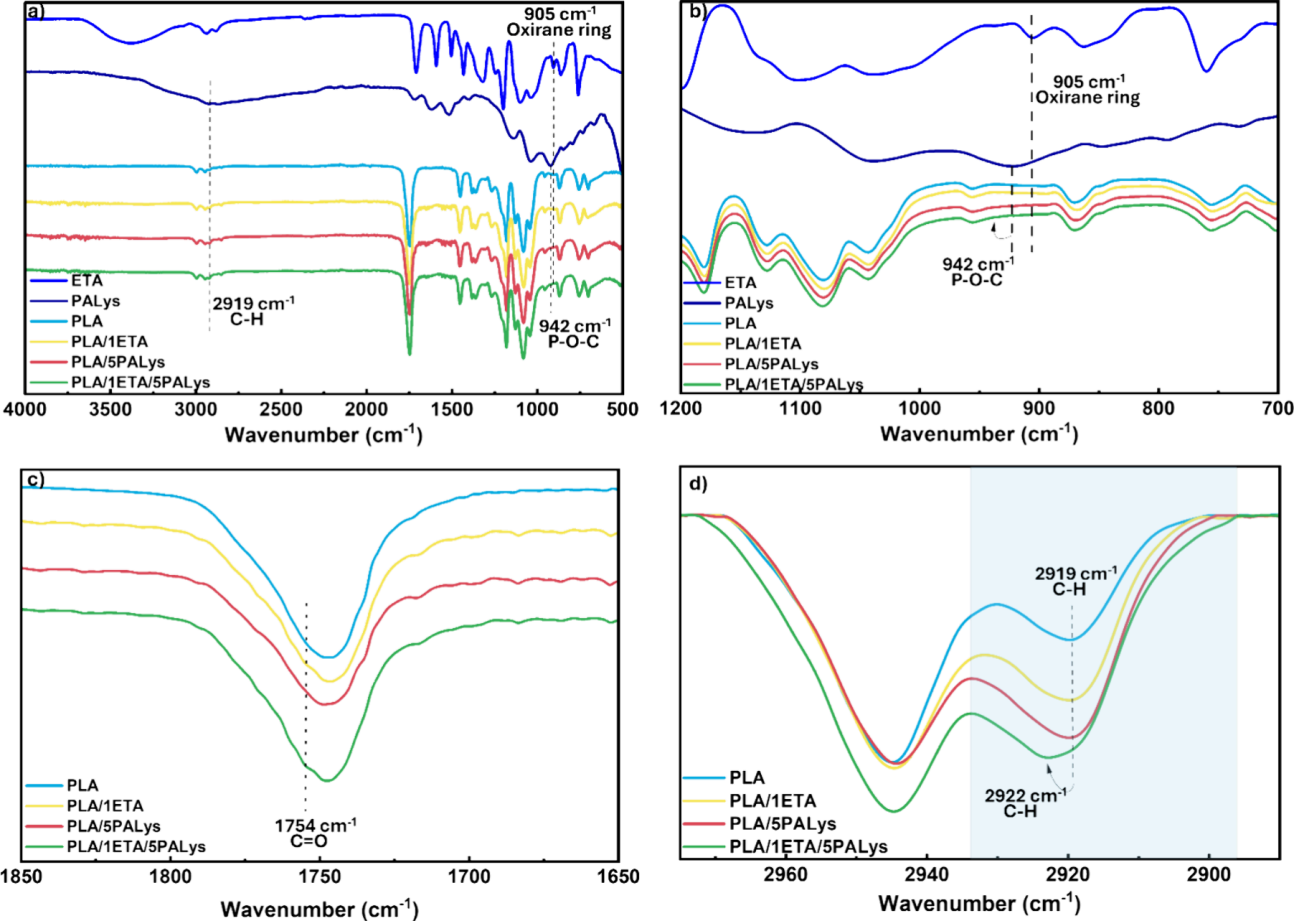
FTIR curves of ETA, PALys, PLA, and modified PLA at (a) 500–4000 cm^–1^, (b) 700–1200 cm^–1^, (c) 1650–1850 cm^–1^, and (d) [baseline-corrected] 2890–2980 cm^–1^ ranges.

### Thermal Stability of PLA Composites

Determining the thermal stability of a polymer is important to identifying the thermal limits to which it could be used. Figure 3 presents the TGA curve for PLA and its composites, and Table 2 reports the TGA values. Pure PLA starts to decompose around 320 °C with a maximum thermal decomposition temperature (*T*_max_) of 346 °C. Moreover, the peak weight loss rate was ascertained as 40.69 wt %/min. This shows that PLA is highly unstable when temperatures reach ∼340 °C, which causes it to decompose rapidly, oxidize, and consume the material.

**Figure 3:**
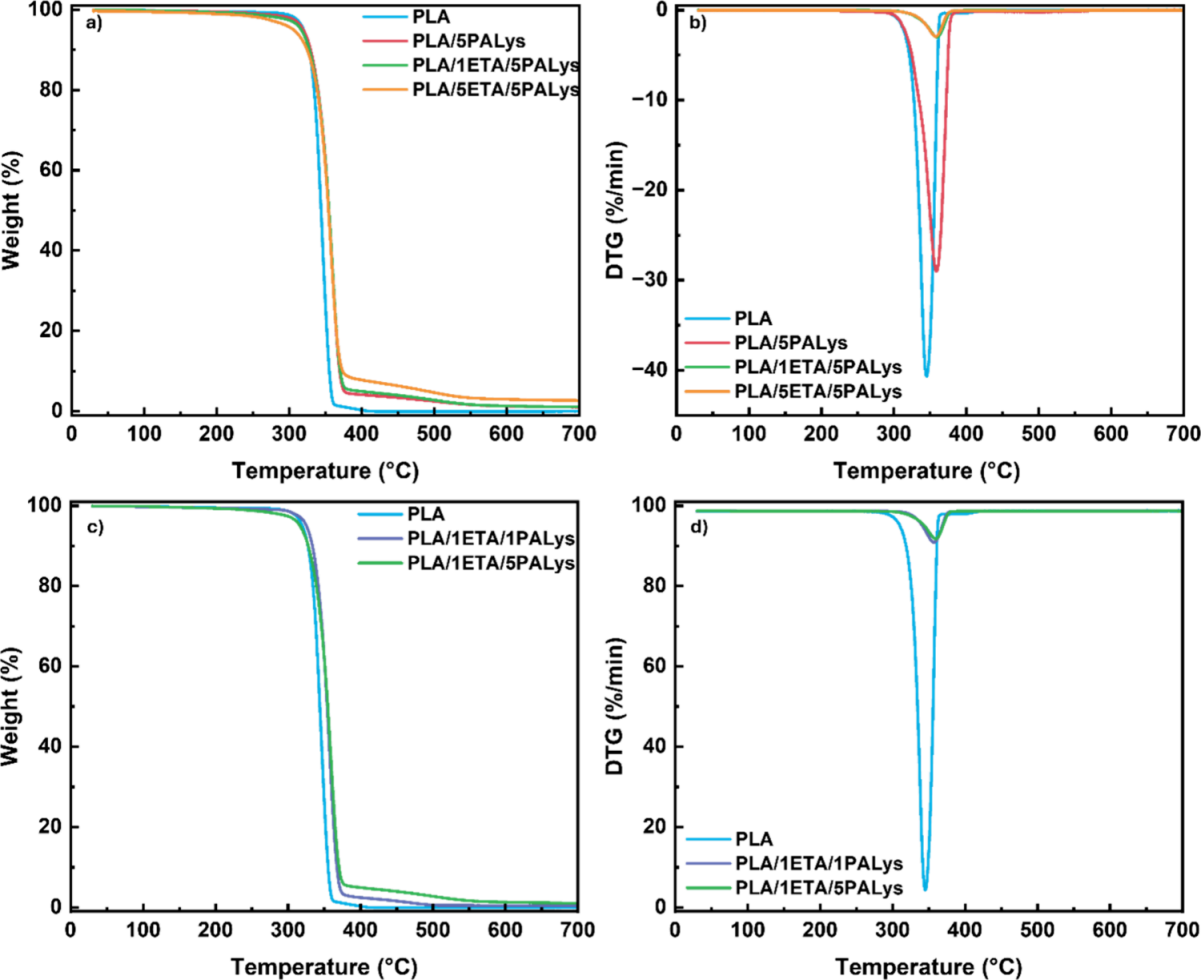
TGA curves for PLA and its composites: (a) weight loss and (b) DTG with varying ETA concentration, (c) weight loss, and (d) DTG with varying PALys concentrations.

**Table 2:** TGA Results for PLA and Its Composites

	°C		wt %	wt %/min
sample	*T*_5%_	*T*_max_	residual at *T*_385 °C_	d(wt %)/d*t*
PLA	320	346	0.76	40.69
PLA/5PALys	319	358	4.37	29.01
PLA/1ETA/1PALys	325	357	2.80	3.36
PLA/1ETA/5PALys	316	359	5.29	2.99
PLA/5ETA/5PALys	306	358	8.38	2.92

The initial decomposition temperature (*T*_5%_), the temperature at which mass loss is 5%, of PLA decreases from 320 to 306 °C as the ETA concentration increases to 5 wt %, as shown in Table 2. The TA compound of ETA decomposes earlier (∼230 °C) than PLA because of the breaking of ester bonds of its outer rings, followed by the decomposition of rigid aromatic rings at 320–384 °C.^25^ As a result, the onset of degradation broadens as more ETA is present in the composite (refer to Figure 3a).

In terms of the rate of decomposition, Table 2 shows that the addition of 5 wt % PALys (PLA/5PALys) has helped decrease the rate of decomposition of PLA by 27.5%. As a result, 4.37 wt % of residue was recorded at 385 °C, the temperature at which decomposition of PLA ceases, as seen in Figure 3a. It demonstrates the capability of PALys to slow down the thermal decomposition of PLA by promoting char formation, a process catalyzed by the phosphorus content of PALys. The addition of ETA has further reduced the weight loss rate from 29.01 to 2.99 wt %/min for PLA/5PALys and PLA/1ETA/5PALys, respectively. ETA is a known carbon source because of the presence of 1,2-benzene diol and 1,2,3-benzene triol in its inner and outer ring, respectively. During thermal decomposition, these gallic acid units undergo rapid dehydration and carbonization from the acid produced by PALys from the pyrolysis of PA.^41^ Hence, it produces char, which stabilizes the thermal decomposition. This is evident with the increase in residue at 385 °C due to the reduction of the weight loss rate at this temperature,^16^ wherein the residue increased from 4.37 to 5.29 wt % for PLA/5PALys and PLA/1ETA/5PALys, respectively. Further increasing the ETA concentration to 5 wt % increases the residue to 8.38 wt %, but it no longer influences the rate of decomposition. It supports the earlier idea that char is forming, but it has reached a plateau that could be an indication of a critical concentration of ETA in the PLA composite.

Besides the reduction in the weight loss rate, Figure 3b also illustrates a shift of the DTG curve to the right. It shows that the maximum thermal decomposition temperature of PLA/5PALys is higher (358 °C) compared to PLA. This shows the effectivity of phosphorus as a char formation catalyst. The addition of ETA (PLA/1ETA/5PALys), however, does not affect PLA *T*_max_. Instead, it changes the weight loss rate at the beginning of the polymer decomposition. It was observed that the weight loss rate has gradually increased for composites that contain ETA, which broadens the DTG curve. A similar behavior was observed for PLA/5ETA/5PALys. This can be associated with the synergistic effect of having an aromatic carbon source in ETA and a P–N FR, wherein the char formation was enhanced by the presence of an additional carbon source. As a result, thermal stability at temperatures between 300 and 359 °C has further improved.

Figure 3c establishes the importance of optimizing the ETA and PALys content. At a low concentration of PALys (1 wt %), the onset degradation temperature is higher (325 °C) compared to a pure sample (320 °C), but its decomposition window is narrow (Δ*T*_degradation_ = 60 °C). It indicates that an insufficient amount of PALys can initially protect the composite from degradation, possibly from gas dilution, but the phosphorus content is inadequate to produce char. This was confirmed by the residue left at 385 °C. The mass loss rate, however, remains low because of the presence of the ETA rings, as presented in Figure 3d.

### Microscale Combustion Calorimeter Results

The MCC results were used as the preliminary screening of the flame retardancy of the sample. It can also provide insight into the flame-retardant pathway, whether it is chemical or physical mechanism-dominant, when analyzed in conjunction with cone calorimetry.^42^ Table 3 depicts the MCC results of PLA and PLA modified with ETA and PALys. The addition of 5 wt % PALys has lowered the pHRR of PLA from 449 to 425 W/g, but the addition of 1 wt % ETA has further reduced the pHRR to 407 W/g. This suggests that ETA can work synergistically with PALys to improve the FR capabilities of PLA. However, increasing the concentration of ETA to 5 wt % showed minimal improvement of the pHRR, which implies that 1 wt % of ETA is enough to amplify the efficiency of the FR system. It was also observed that modifying PLA with ETA but without PALys does not have any significant reduction in pHRR. However, the addition of 1 wt % PALys increased the pHRR of PLA from 449 to 467 W/g, which can imply that the ratio between ETA and PALys is not enough to activate synergistic effects. ETA is a large molecule that is also a good carbon source; nonetheless, it is imperative that there is enough phosphorus content to produce an acidic environment and catalyze the carbonization process of both TA and PLA. This was proven with the addition of 5 wt % PALys, which reduces the pHRR from 467 to 407 W/g.

**Table 3:** MCC Results for PLA with Different ETA and PALys Concentrations

Sample	pHRR (W/g)	THR (kJ/g)	*T*_max_ (°C)
PLA	449	16.45	392
PLA/1ETA	438	16.97	391
PLA/5PALys	425	16.60	394
PLA/1ETA/5PALys	407	16.13	393
PLA/1ETA/1PALys	467	16.87	395
PLA/5ETA/5PALys	406	15.63	395

### Inherent Fire Properties of PLA and Its Composites

The determination of inherent FR properties is important to provide a holistic representation of a polymeric FR. Table 4 presents the inherent flame-retardant properties of PLA and its composites. The LOI value provides the minimum oxygen concentration needed to sustain a flame. Pure PLA has an LOI of 22%, which is a little above the oxygen concentration in air. As a result, they are still susceptible to combustion. However, the addition of PALys increases the LOI value to 30%, which can be associated with charring and dripping. PLA/1ETA/1PALys has a lower LOI (27%) compared to that of PLA/5PALys. It demonstrates the inefficiency of low-concentration PALys to catalyze char formation and effectively utilize ETA as a carbon source because no char was formed and dripping ensued. Increasing the PALys concentration (PLA/1ETA/5PALys) provides sufficient phosphorus content to dehydrate ETA and PLA that catalyzed charring and diluted the gas phase, which resulted in an LOI value of 34%. This shows that the PLA/1ETA/5PALys FR system requires a high concentration of oxygen gas to produce a continuous flame.

**Table 4:** LOI and UL-94 Results for PLA and Its Composites

sample	ave. *t*_1_ (s)^a^	ave. *t*_2_ (s)^a^	dripping	cotton ignition	UL-94 rating	LOI (% vol)
PLA	8.2	9.5	Y	Y	V-2	22
PLA/5PALys	2	1.2	Y	N	V-0	30
PLA/1ETA/1PALys	6.3	0	Y	Y	V-2	27
PLA/1ETA/5PALys	0.3	0	Y	N	V-0	34

a Average of 5 UL-94 bars.

The UL-94 test is another combustion test that provides information about the ability of the material to self-extinguish and its tendency to be a secondary source of ignition. Images of the samples after the first and second ignition are shown in Table S2. Pure PLA passed the V-2 rating despite producing flaming drips that ignited the cotton. PLA has low melt viscosity, which results in melt flow when exposed to an open flame. This produces a flaming drip after the first ignition and a stream of burning molten polymer after the second ignition, which ignites the cotton. Consequently, these flaming drops also reduce the heat conducted through the polymer, allowing it to self-extinguish upon removal of the open flame. The addition of 5 wt % PALys (PLA/5PALys) extinguishes the flaming drops before hitting the cotton. After being dripped, the polymer absorbed enough energy to activate PALys and effectively dehydrate the remaining polymer. Subsequently, char formation occurred as it was deposited on the cotton. This eliminates PLA’s capability of becoming a secondary source of ignition, allowing it to pass the V-0 rating. Moreover, its ability to dilute the gas phase also reduces the average time the polymer is burning after the first flame exposure by 75%, and 87% after the second flame exposure. The second flame exposure of PLA/5PALys reduced the burning time because of charring after the initial flame exposure. The char layer effectively reduced the heat absorbed by the polymer, which was easily dissipated by both dripping and the additional char formation. The PLA/1ETA/5PALys composite also passes the V-0 rating with similar observations on the flaming drip self-extinguishing behavior as PLA/5PALys but further improves on the average burning time after the application of an open flame. The average burning times after the first and second flame application were reduced by 96 and 100%, respectively. With a 1% ETA content (PLA/1ETA/5PALys), the burning polymer experiences sudden extinction of flames after the first flame application. It was accompanied by visible molten polymer bubbling, which can be associated with the release of nonvolatile gases. Upon the second application of the flame, the area of the polymer that was exposed during the first application continued to decompose, burn, and drip. However, the flaming drips extinguish before they reach the cotton. Moreover, the drippings on the cotton visibly contained unburned polymer. Hence, the FR successfully prevented PLA from being a secondary source of ignition. Reducing the amount of PALys prevents it from passing V-0, which can be associated with less gas dilution and charring.

### Combustion Performance

Cone calorimeter testing is a comprehensive combustion performance test that provides information about the flame-retardant behavior of a material. Although the results of the cone testing can be interpreted similar to the MCC, more information can be extracted from it, such as time to ignition (TTI), maximum average release of heat emission (MARHE), fire growth rate (FIGRA), and fire growth potential (FGP). These parameters could be utilized to provide a holistic analysis of the material under forced-combustion conditions. Figure 4 illustrates cone calorimeter results of PLA and its composites, while the detailed tabulated values are presented in Table S3. The results for the addition of ETA and PALys in Figure 4a show that an earlier flame-out time was observed under forced combustion. Pure PLA flames out after 1400 s, followed by PLA/1ETA/1PALys at 1085 s and PLA/5PALys at 1055 s and finally PLA/1ETA/5PALys at 700 s. It suggests that the addition of PALys works to limit the flammability of PLA by reducing the fire load through charring. Adding ETA into the system provides an additional carbon source that produces a more stable residual char that prevents further char oxidation, eventually improving the flame-out time of the modified polymer. Hence, the earlier flame-out of PLA/1ETA/5PALys can be associated with better char formation.

**Figure 4:**
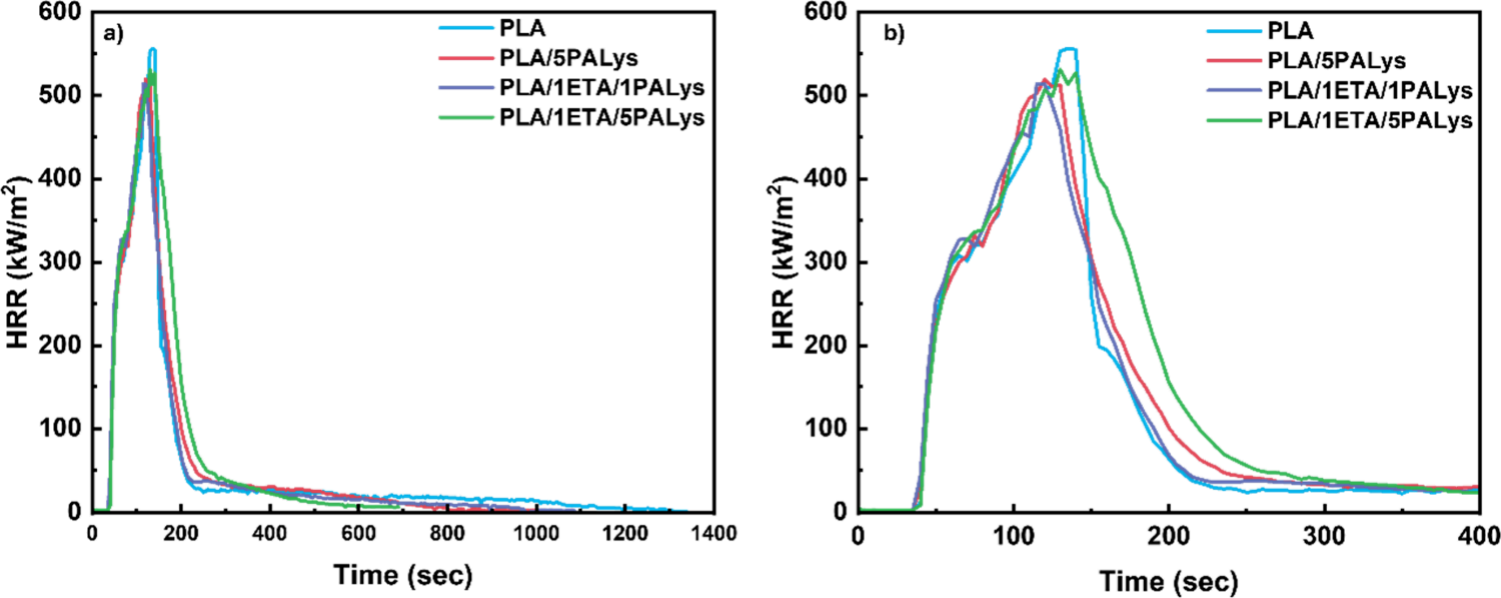
Cone calorimeter results of PLA and modified PLA showing (a) HRR curves with the total burn time and (b) HRR curves that emphasize on the pHRR.

Figure 4b emphasizes the pHRR values of each sample. A reduction in pHRR (∼6%) compared to PLA and increased charring compared to PLA, PLA/5PALys, and PLA/1ETA/1PALys were observed. Despite the excellent inherent fire-retardant properties identified, PLA/1ETA/5PALys has a similar pHRR to pure PLA, but it has undergone char formation, which resulted in an earlier flame-out time. The minor reduction of the pHRR could be attributed to the low melt viscosity of the PLA composite. The study of Karlsson et al.^43^ have shown that the melt viscosity is an important parameter in preventing the transfer of volatile materials to the surface of the burning polymer. A high melt viscosity is desired to hold gases within the melt, concentrate char formation on the surface, and allow the melt to intumesce and form a convection layer to protect the unburned material underneath.

The pHRR for PLA/1ETA/1PALys is similar to that for PLA/1ETA/5PALys, but the former is still completely consumed after cone testing. It confirms that the PALys loading is not enough to catalyze the cross-linking of carbon chains and form aromatic linkages. PA-based flame retardants catalyze the charring process by dehydrating the polymer and the ETA backbone. However, a low concentration of PALys is not enough to catalyze this process for two possible reasons. The first is the nature of the environment: an acidic environment is required for charring to occur. A low PALys concentration has less acidic moieties present in the system, which affects the composites' charring ability. The second is the ratio between the carbon source and phosphorus content. A relatively low phosphorus content would be consumed faster in the presence of a high carbon source loading, which can result in the formation of an aliphatic char structure that is susceptible to char oxidation. Overall, the amount of PALys is important in producing an effective flame retardant for PLA.

### Flame-Retardant Mechanism

To further elaborate on the results of the cone, it is imperative to investigate the behavior of the FR system during combustion. It can be divided into two different phases: the condensed phase and gas phase. For the former, the char formation and physical barrier effect were explored. Pure PLA has no capability to form char because of the fast thermo-oxidative degradation it experiences, which burns through all the material. The addition of 5 wt % PALys (PLA/5PALys) facilitates carbonization of the polymer chain that produces char, as shown in Figure 5a, because of the phosphorus groups present in PALys.^44^ However, it is evident in Figure 5b–d that the char layer is porous. As a result, this is an ineffective protective layer. This can be attributed to the polymer’s low melt viscosity. Based on Figure 5a, most of the PLA/5PALys has burned and the rest has flowed to the corners, which confirms that the melt viscosity was low. Similarly, PLA/1ETA/5PALys exhibits a lessened polymer migration to the corners of the sample holder, an indication of increased melt viscosity, as shown in Figure 5e. Nonetheless, PLA/5PALys and PLA/1ETA/5PALys both left behind char after burning. Interestingly, they have different morphologies as shown in Figure 5b–d,f–k. The latter formed a less porous and more continuous phase of char (refer to Figure 5h,k). For both cases, the melt strength was not enough to hold the gases and prevent their escape to the surface during burning.

**Figure 5:**
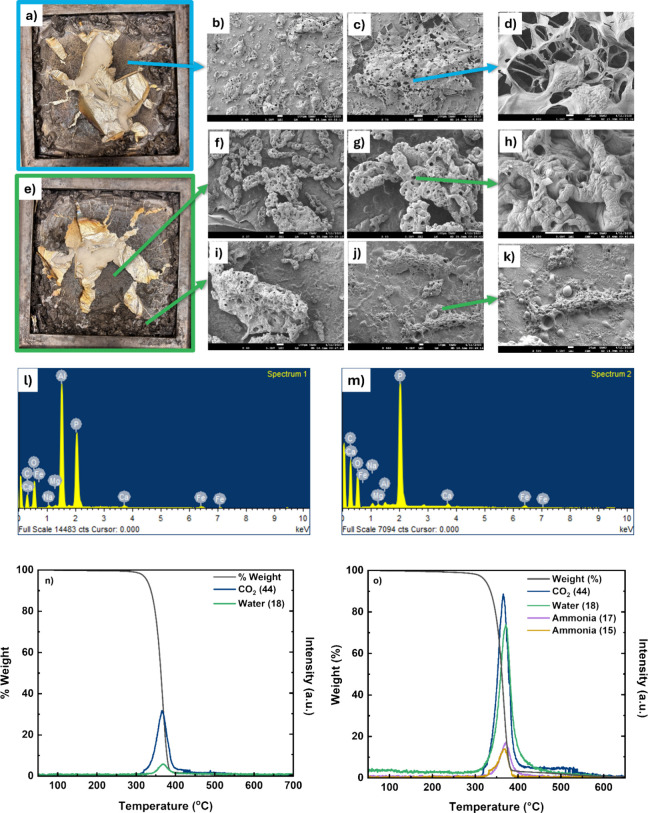
Char images of (a) PLA/5PALys and (b) PLA/1ETA/5PALys after cone testing; SEM images of PLA/5PALys char at the middle with (c) ×45, (d) ×75 (scale bar, 100 μm), and the char structure at (e) ×650 magnification (scale bar, 10 μm); SEM images of PLA/1ETA/5PALys char at the middle with (f) ×37, (g) ×85 (scale bar, 100 μm), and the char structure at (h) ×250 magnification (scale bar, 10 μm); SEM images of PLA/1ETA/5PALys char at the corner with (i) ×60, (j) ×350 (scale bar, 100 μm), and the char structure at (k) ×500 magnification (scale bar, 10 μm); EDS elemental analysis for (l) PLA/5PALys and (m) PLA/1ETA/5PALys; TG-MS curve for (n) PLA and (o) PLA/1ETA/5PALys.

Another factor that can contribute to the FR performance is the quality of the char. The char layer should be a physical barrier between the pyrolysis zone and the unburned material. To be effective, the char formed must be able to withstand oxidation during the burning. Ideally, cross-linked char or an aromatic linkage is desired because of its high bond dissociation energy (BDE) (>450 kJ/mol).^45^ This makes the char stable, allowing better resistance against oxidation and longer protection of the unburned materials. PA-based FRs mainly function at the condensed phase through dehydration, as mentioned earlier. In this process, phytic acid decomposes around 200 °C to generate phosphoric acid that facilitates the carbonization of the polymer matrix.^46^ Figure 5l,m presents the EDS results of PLA/5PALys and PLA/1ETA/5PALys. It can be depicted that both composite residual chars contain phosphorus, which improves the carbonization of PLA during the early stages of decomposition. However, Figure 5l presents a high aluminum peak that is an indication of a thin char layer for PLA/5PALys. The char molecular structure did not undergo enough carbonization to prevent char volatilization due to oxidation. Moreover, the porous structure of the char exposes a larger surface area through which oxygen can diffuse through. As a result, char volatilization becomes more prominent. Conversely, the addition of ETA for PLA/1ETA/5PALys forms more char in the middle and a better overall char structure. This is an indication that ETA was able to increase the melt viscosity, and the presence of aromatic structures contributed to the formation of a stable char.

The gas-phase action of the modified PLA stems from the addition of PALys. Figures 5n and 5o illustrate the TG-MS curves for PLA and PALys/1ETA/5PALys, respectively. It was observed that, for PLA, CO_2_ (*m*/*z* = 44) and H_2_O (*m*/*z* = 18) were the most prominent gas products, as shown in Figure 5n. The addition of PALys into the matrix contributed to the gas-phase action of the FR by releasing ammonia (*m*/*z* = 17 and 15), which is a noncombustible gas, starting at 320 °C and peaking at 370 °C. The NH_3_^+^ groups form ammonia once released from its ionic bond with PA, as shown in Figure 5o. It functions as a diluting agent to reduce the concentration of burnable volatile materials on the surface of the composite. In addition, it was observed that the CO_2_ and H_2_O peaks shifted to the right with an increased intensity. It is an indication of a slow thermo-oxidative degradation signifying that the combination of ETA and PALys has improved the thermal stability of PLA. Moreover, the increase in intensity can be attributed to the catalyzed dehydration and carbonization of both ETA and PLA chains, which release CO_2_ and H_2_O that can aid in diluting the gas phase. It was also observed that the CO_2_ curve does not immediately drop to zero following the its bulk release at 365 °C, which implies that parts of the char undergo volatilization due to oxidation at high temperatures that converts C_aliph_–C_aliph_ bonds into CO_2_.^45^ With this, the observed delay in the bulk release of ammonia could be associated with the gases being trapped within the molten polymer as carbonization occurs. Consequently, it slowly releases the trapped gases in the matrix as the polymer melt continues to expand and eventually collapse due to the low melt strength or the volatilization of char that was initially formed.

Thus, Figure 6 illustrates the proposed FR mechanism of PLA/1ETA/5PAlys. Upon ignition, ETA and PALys FR capabilities are activated and start to affect both the condensed and gas phase. In the condensed phase, char formation was catalyzed by the phosphorus content of PALys. The char is a combination of aromatic and cross-linked structures from the influence of ETA chains and phosphorus catalysis of the PLA backbone. However, parts of the carbonized polymer retain a simple C_aliph_–C_aliph_ bond that is easy to oxidize under extended periods of time, leading to the formation of additional pores in the char. The gas-phase action showed the release of ammonia from the PALys FR beginning at 320 °C. This dilutes the gas phase and reduces the flammable gas concentration. Moreover, more CO_2_ and H_2_O were released, which also absorbs energy and dilutes oxygen concentration. Continuous release of CO_2_ was also observed because of the volatilization of char. It then self-extinguishes and leaves char residues.

**Figure 6:**
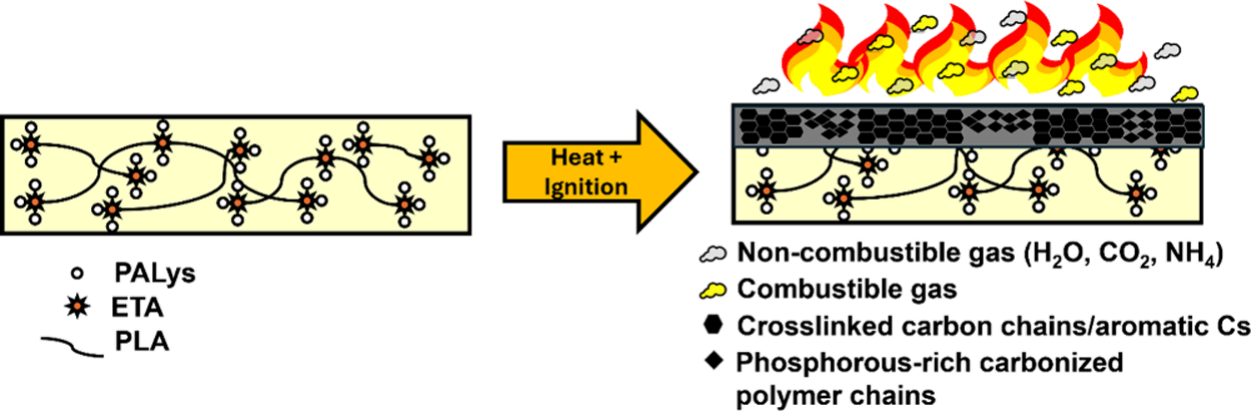
Proposed flame-retardant mechanism of PLA/1ETA/5PALys.

## Conclusions

PLA was successfully modified using ETA and PALys as an effective FR to improve its inherent flame retardancy. ETA bonded with PLA and PALys, which served as an anchor between the polymer chain and primary FR, and provided an additional carbon source. PLA/1ETA/5PALys presented a single broader polymer decomposition curve between 316 and 385 °C with 5.29 wt % residue left at 385 °C, improving the thermal stability of PLA. Moreover, the weight loss rate was reduced by 92.7%. In terms of FR performance, PLA/1ETA/5PALys achieved a UL-94 V-0 rating with decreased flaming drips and reduced burning times of 96 and 100% after the first and second flame application, respectively, compared to PLA. It also obtained a 34% vol LOI value, which emphasizes its excellent inherent FR capabilities. Nonetheless, it can reduce the flame-out time from 1400 to 700 s because of char formation and reduced fire load. The proposed FR mechanism starts with the decomposition of the PLA/1ETA/5PALys at 316 °C, wherein the phosphorus content of PALys starts to breakdown and dehydrate ETA and PLA, catalyzing the carbonization of the polymer and starting the char formation. It is then followed by the release of ammonia at 320 °C that dilutes the gas phase of the combustible material. The material then self-extinguishes with a char residue left after testing. The study showed excellent FR capability by attaching the FR to the polymer end group. With this, it is possible to utilize PLA for long-term applications that require an FR system, such as automotive liners and interior components. In addition, it reduces the need to incorporate high concentrations of FRs and provides flexibility to modify PLA further with other functional additives, such as impact resistance, UV stabilizers, and light stabilizers.

## Supplementary Material



## Notes

Chemical structure of reactants and the possible chemical structure of the polymer after reactive extrusion, additional characterization of modified PLA, thermal stability of bioadditives, and additional flame-retardant performance data (PDF)
