# Supplementary material for: Enhancing the Inherent Flame Retardancy of Polylactic Acid by Anchoring Phytic Acid-Lysine Using Epoxidized Tannic Acid
Source: ACS Appl Polym Mater. 2025 Dec 2;7(23):16217–27. doi: 10.1021/acsapm.5c03450 (PMC12706741; doi:10.1021/acsapm.5c03450)
Supplement: Supplementary file 1 [file ap5c03450_si_001.pdf]

# Supporting Information

# Enhancing Inherent Flame Retardancy of Polylactic Acid by Anchoring Phytic Acid-Lysine using Epoxidized Tannic Acid

Jazmine Aiya D. Marquez, Wan Zhang, Navaporn Suphavilai, Manish Shetty, Hae-Kwon Jeong, Qingsheng Wang *

Artie McFerrin Department of Chemical Engineering, Texas A&M University, College Station, TX 77843, United States

* Corresponding author, email address: qwang@tamu.edu

| Reactant | Structure | Symbol |
|---|---|---|
| PLA | 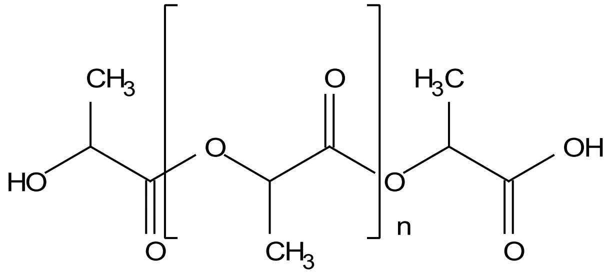 | 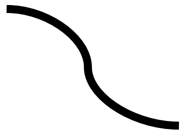 |
| ETA | 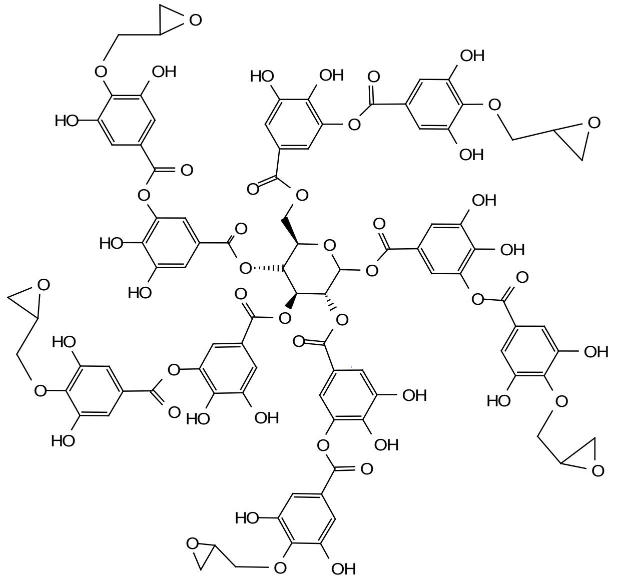 | 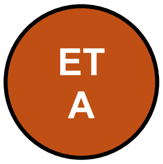 |
| PALys | 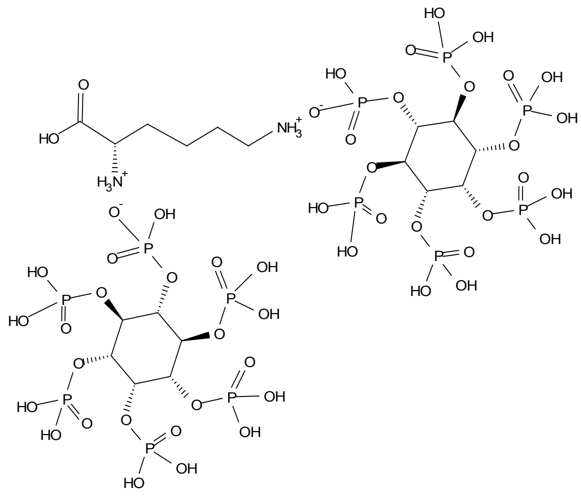 | 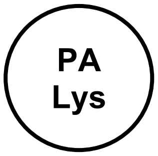 |

**Figure S1.** Chemical Structure of polylactic acid (PLA), epoxidized tannic acid (ETA), and phytic acid-lysine (PALys) salt

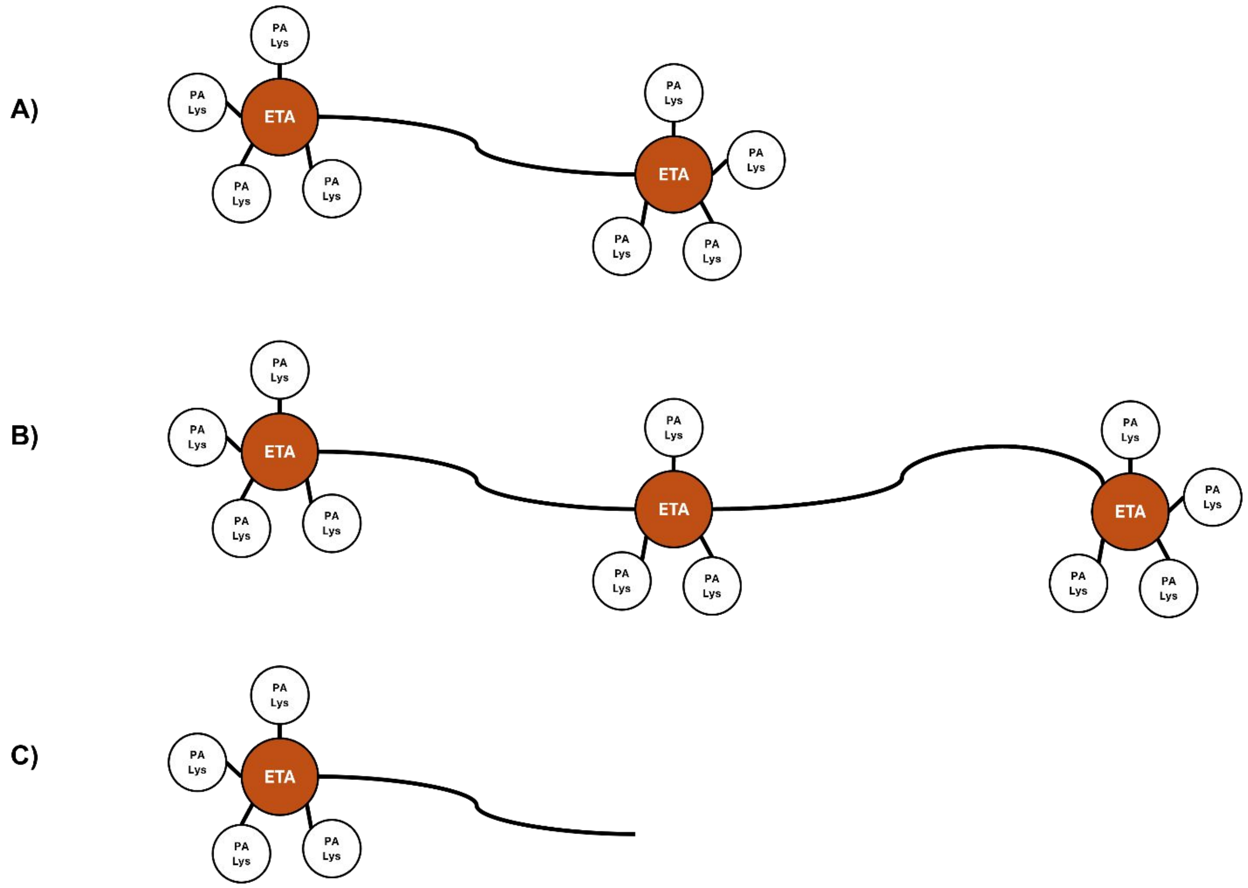


**Figure S2**. Possible Chemical Structure of Polymer after Reactive Extrusion: (A) Dumbbell molecular structure with ETA and PALys at the endgroups, (B) chain extension of PLA chain with ETA connecting two PLA chains and ETA+PALys at the endgroups, (C) Only one end group has reacted with ETA and has PALys

**Table S1**. Quantitative comparison of peak broadening from curve fitting

| | cm$^{-1}$ | | |
|---|---|---|---|
| Sample | FWHM | Centroid | Symmetry |
| PLA | 13.23 | 2920 | Symmetric |
| PLA/1ETA | 15.27 | 2920 | Symmetric |
| PLA/5PALys | 16.50 | 2920 | Asymmetric |
| PLA/1ETA/5PALys | 18.84 | 2921 | Asymmetric |

Where, FWHM – full width at half maximum – a measure of signal resolution by describing the width or spread of a signal

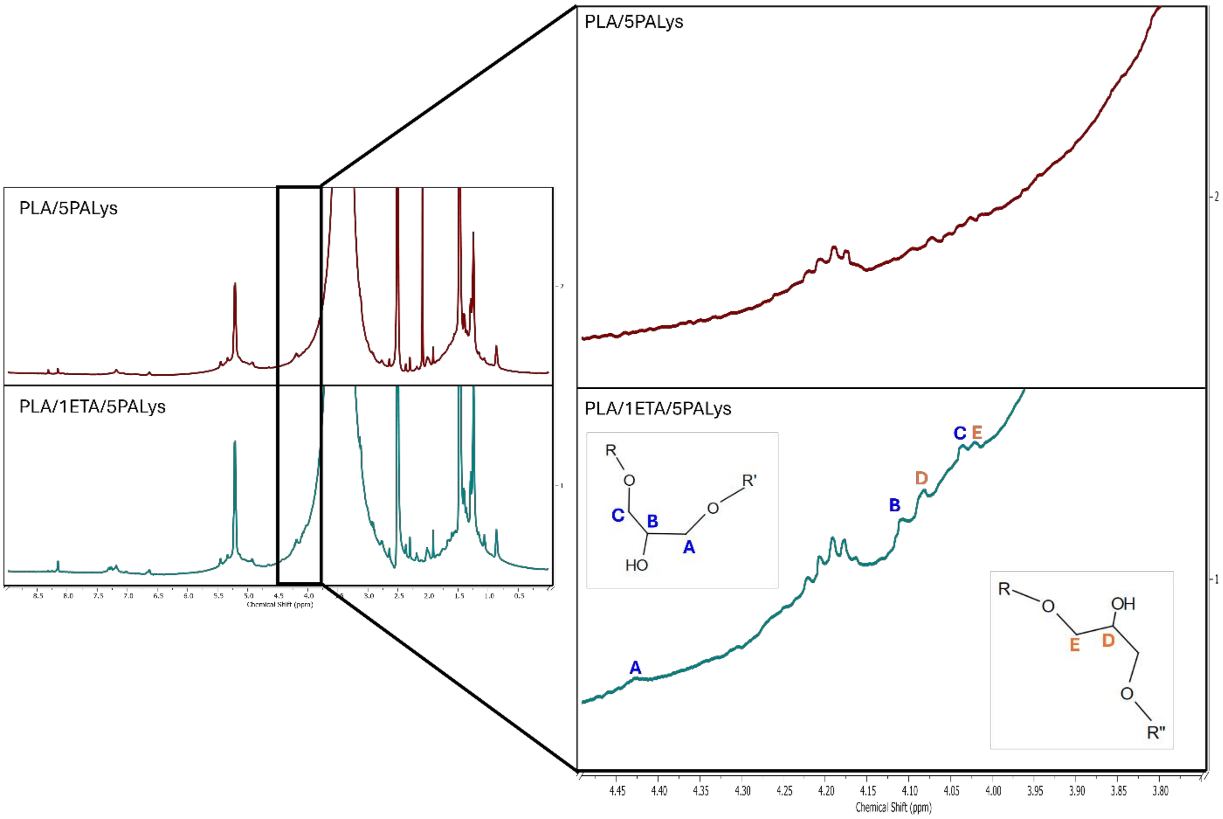


**Figure S3**. 1H NMR of PLA/5PALys and PLA/1ETA/5PALys highlighting the connectivity between PLA/ETA and PLA/PALys through reaction with carboxylic acid end groups, as well as the TA molecule side of the bond between PLA/ETA from the -OH end group, where R is the tannic acid group, R' is PLA (carboxylic acid end group) or PALys carboxylic acid, and R" is PLA (-OH end group).

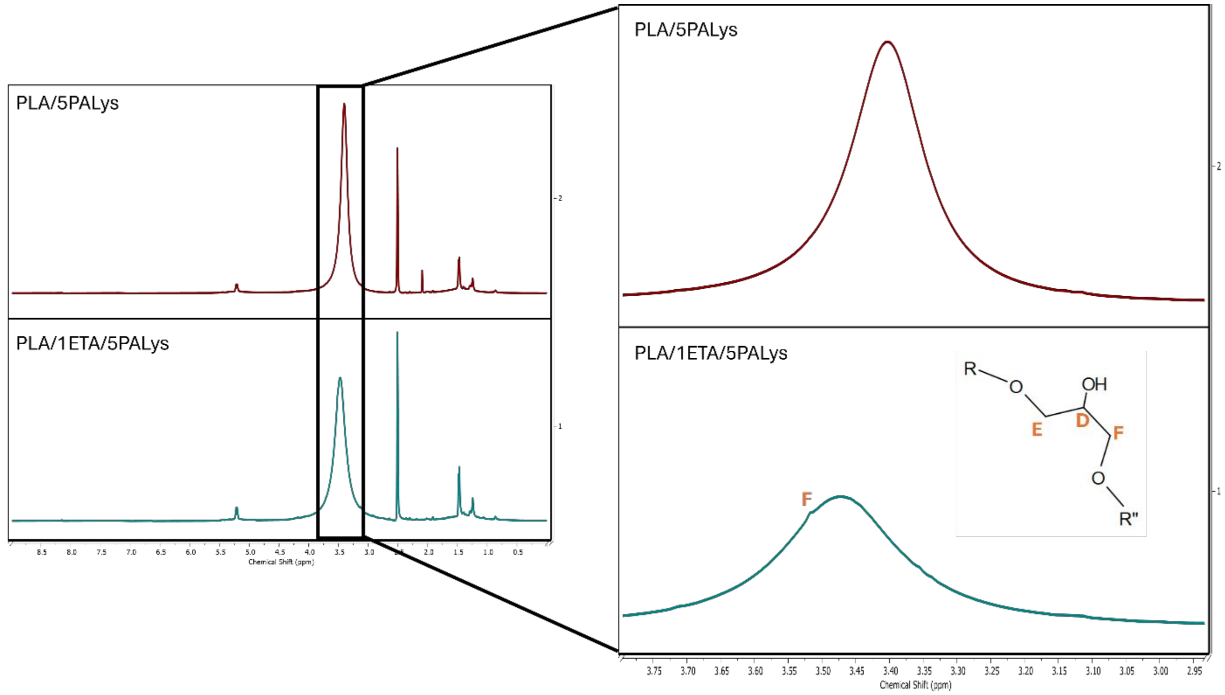


**Figure S4**. 1H NMR of PLA/5PALys and PLA/1ETA/5PALys highlighting the connectivity between PLA/ETA through reaction with -OH end groups which shows the PLA side of the bond between PLA/ETA, where R is the tannic acid group, and R" is PLA (-OH end group).

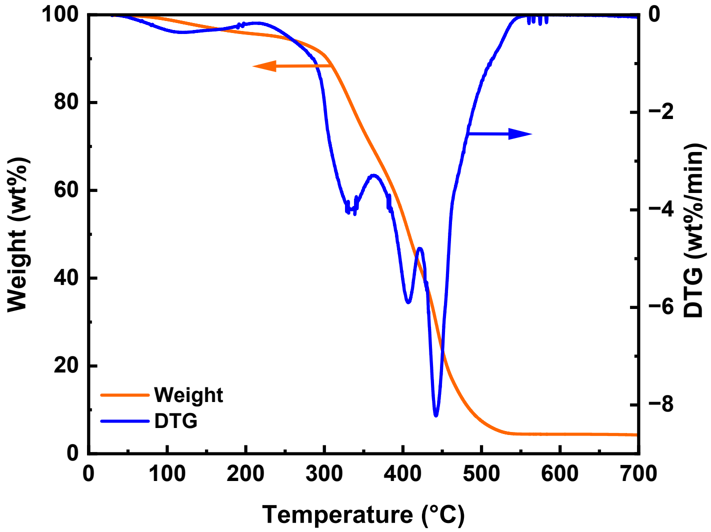


**Figure S5**. TGA and DTG curves of ETA in air with a heating rate of 10 °C/min and a temperature range of 30-700°C

**Table S2.** UL-94 vertical combustion comparison of after flame exposure behavior

| Sample | PLA | PLA/5PALys | PLA/1ETA/1PALys | PLA/1ETA/5PALys |
|---|---|---|---|---|
| | | After $1^{st}$ flame exposure | | |
| Time | | | | |
| 1 sec | 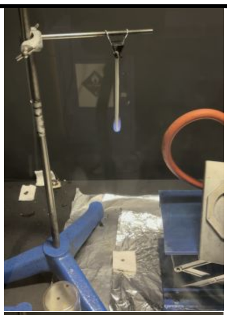 | 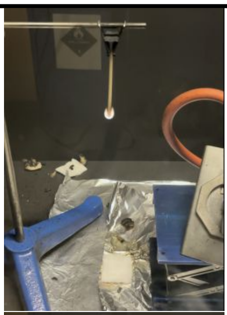 | 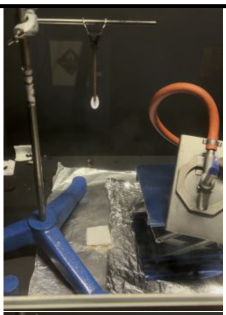 | 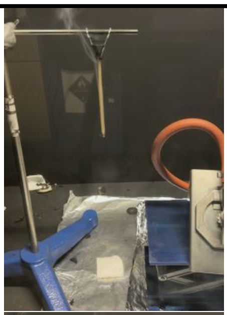 |
| 2 sec | 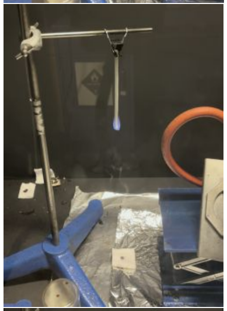 | 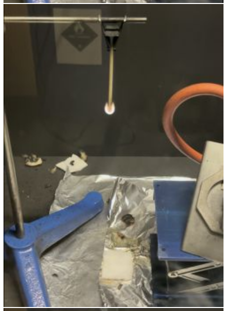 | 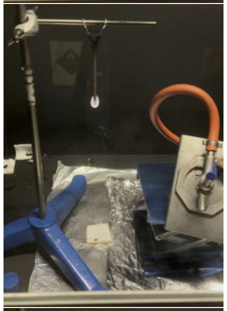 | 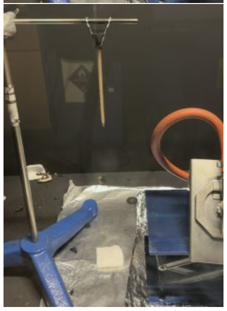 |
| 5 sec | 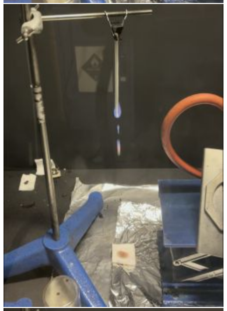 | 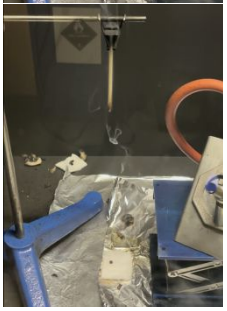 | 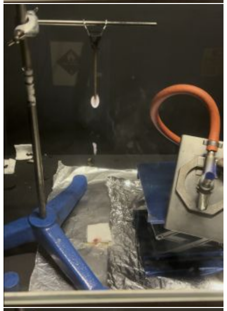 | |
| 8 sec | 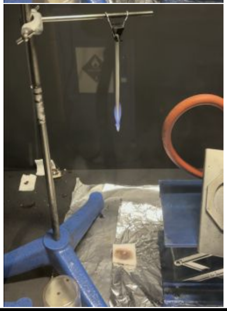 | | 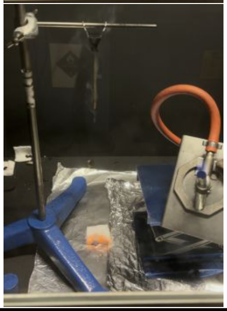 | |

| | | | | |
|---|---|---|---|---|
| 10 sec | 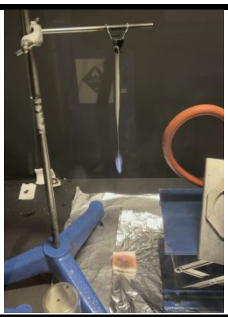 | | | |
| After 2nd flame exposure | | | | |
| Time | | | | |
| 1 sec | 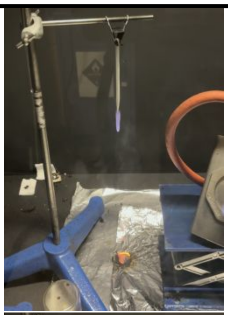 | 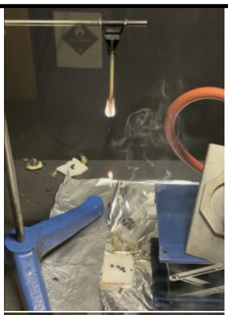 | 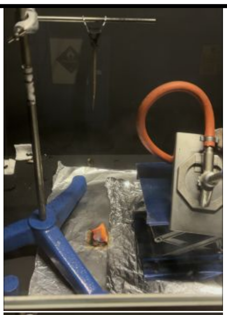 | 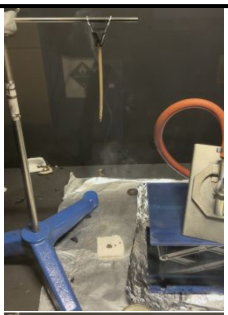 |
| 2 sec | 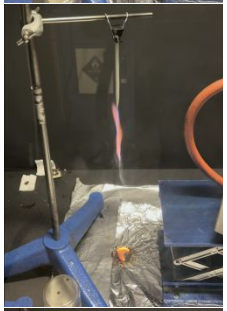 | 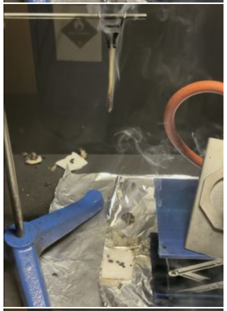 | 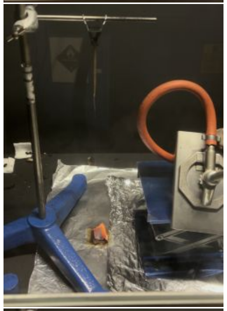 | 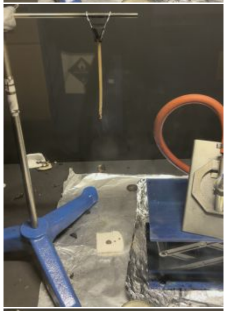 |
| 5 sec | 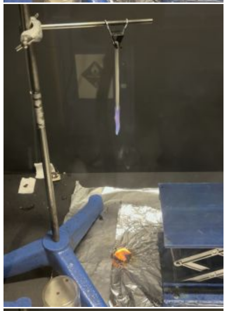 | 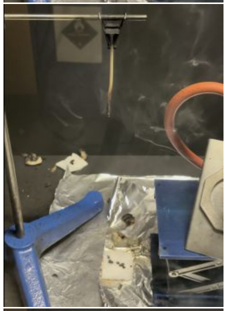 | 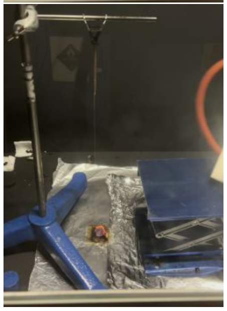 | 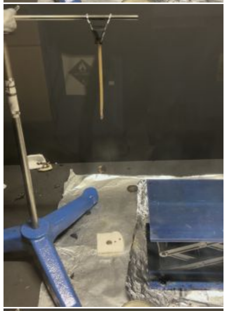 |
| 8 sec | 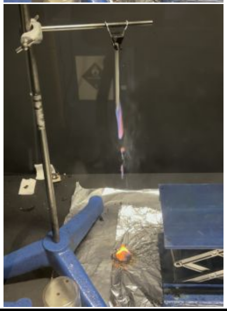 | 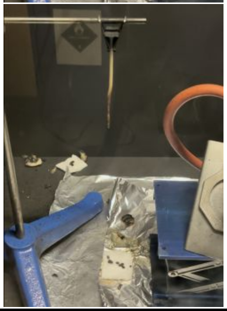 | | 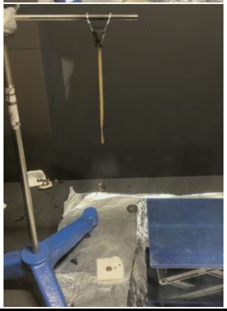 |

| | | | | |
|---|---|---|---|---|
| 10 sec | 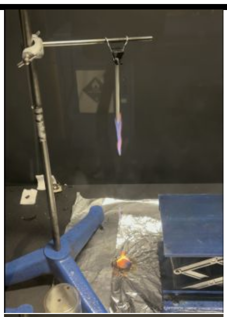 | 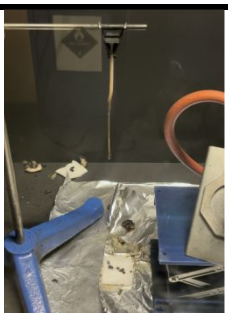 | | 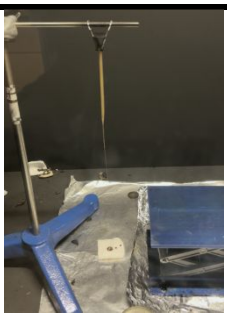 |
| 20 sec | 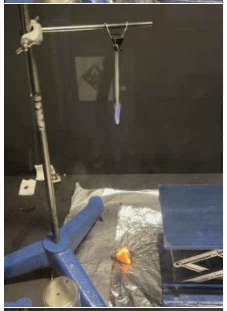 | | | |
| 30 sec | 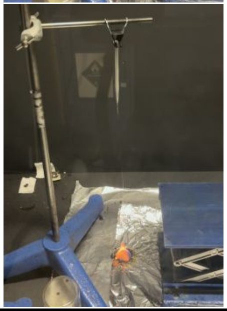 | | | |

**Table S3.** Detailed cone calorimeter results of PLA and modified PLA

| | s | s | $kW/m^2$ | $MJ/m^2$ | $m^2/m^2$ | $m^2$ | MJ/kg | g/s | $kW/m^2$ | $kW/m^2s$ |
|---|---|---|---|---|---|---|---|---|---|---|
| Sample | TTI | $t_{pHRR}$ | pHRR | THR | TSR | TSP | pEHC | pMLR | MARHE | FIGRA |
| PLA | 39 | 135 | 556 | 67.6 | 0 | 0 | 77.9 | 0.2970 | 276 | 4.12 |
| PLA/5PALys | 38 | 120 | 519 | 65.6 | 0 | 0 | 78.2 | 0.2759 | 271 | 4.33 |
| PLA/1ETA/1PALys | 37 | 115 | 514 | 62.4 | 0 | 0 | 79.1 | 0.3289 | 270 | 4.47 |
| PLA/1ETA/5PALys | 39 | 130 | 531 | 69.9 | 0.37 | 0.003 | 79.5 | 0.2775 | 295 | 4.08 |

Where, TTI – time to ignition, $t_{pHRR}$ – time to peak heat release rate, pHRR – peak heat release rate, THR – total heat release, TSR – total smoke released, TSP – total smoke production, pEHC – peak effective heat of combustion, pMLR – peak mass loss rate, MARHE – maximum average rate of heat emission, FIGRA – fire growth rate average
